# Sierra Leone laboratory systems – now and future

**DOI:** 10.4102/ajlm.v5i3.549

**Published:** 2016-10-31

**Authors:** Isatta Wurie

**Affiliations:** 1 African Society for Laboratory Medicine, Addis Ababa, Ethiopia; 2 Association of Public Health Laboratories, Silver Spring, Maryland, United States

## Introduction

The impact of the 2013–2016 Ebola virus disease outbreak in the Mano River Union region, which includes Liberia, Sierra Leone and Guinea,^1^ is a direct indicator of the acute limitations in national healthcare systems and the critical role played by diagnostic and public health laboratories. Despite having a Central Public Health Reference Laboratory, Sierra Leone lacked the capacity to provide the Biosafety Level 3 laboratory support that is required to effectively direct responses and that could have offset such a prolonged and costly health systems war against the outbreak.

Before the outbreak, Sierra Leone had in place both a national Medical Laboratory Policy and a five-year strategic plan for 2010–2015,^2,3^ and had achieved some notable successes. These included the establishment of the first Laboratory Medicine/Science undergraduate degree course to increase the human resource pool of laboratorians, the development of national norms and standards to inform laboratory support for the national health package, and the activation of the Central Public Health Reference Laboratory. The Central Public Health Reference Laboratory formed the apex to institute a public health laboratory network to diagnose key national priority diseases, such as influenza, yellow fever and measles, support the restructuring of laboratory tier systems, and support the integration of surveillance activities, especially for HIV molecular testing and the national health demographics health survey. However, during the implementation of the strategic plan, the country experienced two major disease outbreaks – cholera in 2012^4^ and Ebola virus disease in 2013^1^ – that crippled the implementation of other key interventions.

Sierra Leone is now focused on building a resilient healthcare delivery system. This will require major changes to improve routine services at clinical and public health laboratories throughout the country. The strategy is to develop comprehensive multi-programme integration, including tuberculosis, malaria and HIV, as well as disease surveillance and preparedness combined with best practices for successful healthcare delivery adapted to suit the country’s infrastructure, capability and environment.

## Going forward: From now to the future

For laboratory services to reach the goal of supporting an improved healthcare system, the following restructuring and strengthening framework platform is being developed, where it is lacking, or in an accelerated and strengthened implementation mode, where it existed.

### Leadership

In the reconstruction of a national healthcare laboratory system, two key components are: (1) political will at the Ministry level of the national government; and (2) health-sector technical leadership to motivate and drive the concepts and importance of laboratories in healthcare delivery. Using the Ebola virus disease outbreak as an example, there were 16 international laboratories supporting the outbreak response. Coordinating the activities of these partners was a major challenge, because the government did not have a defined leadership plan for dealing with such competing issues. Therefore, the operations of the international laboratories were independent, with all but one having an exit plan via training of local experts.

Moving forward, the government has put in place a service-level agreement that ensures health-sector commitments and coordinated support for laboratory services. First, political will was secured by including laboratory service indicators for improved health into the Health Ministry’s development goals. Next, a directorate or unit charged with policy direction and monitoring under the office of the Director of Hospital and Laboratory Services is being strengthened, with more staff linking with Directorate of Health Systems Strengthening. To manage integration of clinical and public health laboratory and disease surveillance services, operational leads will define the strategic scope of work for key professional positions through the health commission. Finally, a team of specialists in laboratory science, process management, laboratory-related programmes, epidemiology, surveillance and health development partnership will be established to provide technical oversight in an advisory capacity, advocate for laboratory resources and act as monitors to ensure an integrated approach is taken toward achieving the national health development goals.

### National direction

#### Health sector guidance tools

Laboratory services form an integral part of quality healthcare planning and delivery. Although national documents existed in Sierra Leone,^2,3^ their integration and coordination was limited. With the restructuring of departments within the Ministry of Health and Sanitation, the Health Systems Strengthening unit instituted and with emphasis by the Director of Hospital and Laboratory Services recognises that reliable, integrated and well-structured laboratory services are essential if the national Basic Package of Essential Health Services delivery is to be achieved. This Health Systems Strengthening–Director of Hospital and Laboratory Services combination is now charged with developing and consolidating tools and processes to guide health sector reconstruction and development for a strengthened and resilient health service.

The health sector guidance tools comprise six essential elements: (1) the Basic Essential Health Package defines the level of laboratory support required at all tiers of the healthcare system and was developed with community-based input taken into consideration; (2) the National Laboratory Policy defines the scope of operations of laboratories within the mandate of the health sector; (3) the National Laboratory Strategic Plan translates the policy into actionable services through strategic interventions; (4) the National Integrated Disease Surveillance Plan specifically defines the responsibility of laboratory support in the areas of public health, outbreak response and epidemic monitoring; (5) the National Implementation framework and log plan outlines the annual plan that will provide the basis for smart monitoring and evaluations; and finally, (6) the Laboratory Operations Standards define the minimum standards required for laboratory functions to support clinical and public healthcare facilities.

### Functional health sector laboratories

#### Defining functional laboratory systems

The function of a laboratory system is defined and tailored according the health needs of a particular country, taking into account regional and other international health regulations. The overall aim is to reduce mortality and morbidity through prompt diagnosis for effective treatment, protection of the community from outbreaks by giving special focus to select, epidemic-prone diseases relevant to the country, ensure efficiency of services with appropriate laboratory testing and, above all, ensure the confidence of patients, thereby facilitating positive health-seeking behaviour. A structured and organised laboratory service will strive to ensure staff are motivated, thereby improving quality staff recruitment and their retention.

#### Core laboratory services

For Sierra Leone, core laboratory services were recommended based on their role in quality laboratory service delivery in the context of the entire healthcare system. The first and most obvious is diagnosis. Clinicians require accurate and timely laboratory results to inform appropriate patient management and monitor treatment outcomes. Thus, the clinical services to be offered through Sierra Leone’s network of clinical facilities and across all tiers of the national healthcare system will be selected for their ability to improve diagnostic accuracy, reduce misdiagnosis and provide prompt intervention. Services associated with surveillance will be recommended based on their capacity to provide effective disease carrier identification, contact tracing and linkages with inter-sectorial integrated interventions. Treatment services will be more targeted, with the goal of using drugs selectively and thus minimising potential antimicrobial resistance, in addition to providing effective monitoring of treatment progress and side effects. Other factors that are considered include the role of services in public health, namely, monitoring of epidemic trends and early diagnosis of pathogens to support containment and infection prevention. Health assessments and screening capabilities are also key factors, as constant monitoring of key populations, such as pregnant women and infants for wellness status or potential referral and screening of donor blood, are critically important. Finally, support of a laboratory information system is crucial to providing empirical data for assessment of risk factors and monitoring the general health of the country’s population through prevalence and incidence indicators.

### Sustaining laboratory operations

#### Minimum laboratory standards

Governance and leadership coordination are also required to push the daily operations of laboratories through a coordinated allocation of appropriate resources to meet the minimum standards necessary for the functioning of clinical and public health laboratories. The standards and the priority levels set for their implementation in Sierra Leone, through its National Health Sector Strategic Plan 2015–2020,^5^ are depicted in Table 1.

**TABLE 1:** Minimum laboratory standards and implementation priorities in Sierra Leone, 2016.

Priority Level	System	Core Structures	Support
LEVEL 1	Laboratory administration	Infrastructure: logical spatial reorganisation of laboratory	Building or defined laboratory space
		Utilities	24-hour energy
			Quality water
		Equipment	Maintenance/service contract; appropriate and affordable equipment and supplies
		Stocks and inventory management	Supply chain management
		House keeping	Maintenance logistics
			Site security
	Human resources	Service levels	Career path
			Recruitment plan
			Retention
		Training	Curriculum (short /medium and long term)
			In-service
			Pre-service
			FELTP
			Targeted (tailor made)
	Health and safety	Safety officials	Policy and manual
Code of practice
		Biosafety and biosecurity	Specimen management and archiving
LEVEL 2	Data management	Laboratory Information Management System	Integrated electronics
LEVEL 3	Research and development	Ethics and coordination unit	Linkage to Health Systems Strengthening and Planning unit
	Surveillance and coordination	Integrated strategic plan	Rapid Response Manual
LEVEL 4	Total quality management	Quality assurance	Implementing SLMTA

FELTP, Field Epidemiology and Laboratory Training Program; SLMTA, Strengthening Laboratory Management Towards Accreditation.

**FIGURE 1:**
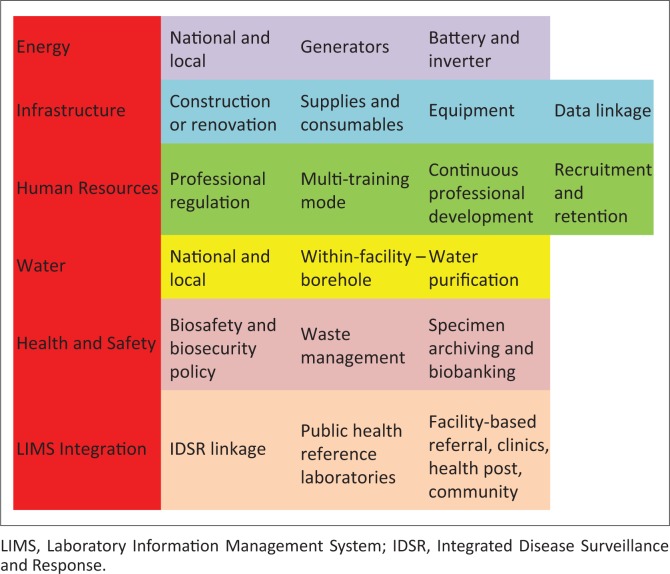
Sustainable reconstruction framework.

To institute these basic minimum standards for laboratory operations, reconstruction of Sierra Leone’s laboratory system is being coordinated using a multi-faceted approach to ensure that all possible alternatives are explored (Figure 1).^6^ Ultimately, more than one approach may be required to achieve effective services. For example, to ensure adequate water supplies, both national and local efforts will be needed. Solutions may vary by location, where purification of a surface water source may be practical for a low-volume remote laboratory, but drilling a new well or a bore hole with filter action treatment may be required for a high-volume, referral laboratory.

Finally, in order for a laboratory system to be truly sustainable, it is important to define and allocate resources for ‘hidden’ costs. Hidden operational costs are easily overlooked because of more obvious and tangible costs related to diagnostic capacity. However, it is important to realise that the entire function of a laboratory is dependent on these costs. For example, housekeeping, security, caretaking, cleaning and building maintenance are all necessary and important general functions that must be maintained. Similarly, infrastructure costs, such as communications, utilities and waste disposal, are important for general functioning of the laboratory, and staffing and training-related costs, such as career development, staff workload ratios, training and continuing education, are critical for staff proficiency and retention. More obvious are equipment and supply-related costs, such as service contracts, inventory systems, safety compliance and certifications.

### Public health laboratory emergency preparedness

During public health emergencies, laboratory services are a critical component of the response for directing timely actions and interventions. Through the provision of empirical data, laboratories set the pace to confirm diagnosis and support the testing of epidemiologically-significant specimens with potential public health implications.

**FIGURE 2:**
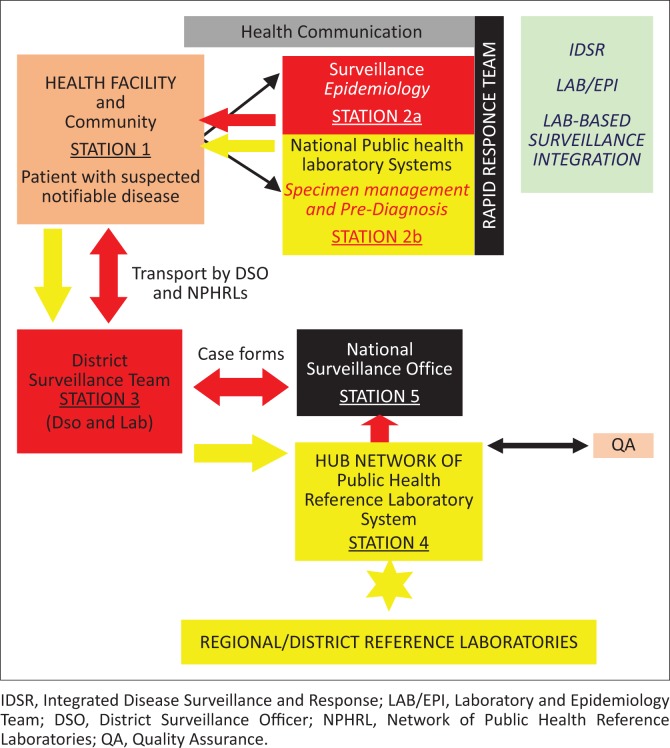
Framework for the reconstruction of laboratory system preparedness.

To achieve the objective of prompt response to contain an outbreak, laboratories in Sierra Leone are part of an integrated national Rapid Response Core Team (Figure 2). The Rapid Response Core Team works with surveillance experts, health communicators, clinicians and zoonotic experts in the investigation and identification of the causative agents of outbreaks. The integrated action flow involves: (1) notification and logistics preparedness; (2) specimen management, which includes collection, storage and transportation of biosamples; (3) testing – and ensuring a quality assured result – at the facility; (4) laboratory-based surveillance and confirmation within the network of public health reference laboratories; and (5) reporting of results for action by clinicians and for public health interventions, and monitoring of trends by surveillance and epidemiology experts.

### Integrated laboratory data management

In Sierra Leone, the Network of Public Health Reference Laboratories Systems network, as part of the National Health Institute or Agency, will serve as the national focal point for capturing the laboratory data that is necessary for monitoring disease trends, ensuring that prevention and control strategies are undertaken properly – activities that also are essential for policy and decision making. In addition, this agency will maintain and communicate laboratory data and information to assist in identification, understanding and controlling of disease outbreaks.

### Conclusion

Although much remains to be done, since the official end of the Ebola virus disease outbreak, progress has been made toward the reconstruction of a sustainable healthcare laboratory system in Sierra Leone. Next steps will involve developing and strengthening partnerships among the countrywide network of laboratories and developing a national map of laboratory resources to assist these partnerships and improve communication and training.
